# Supplementary material for: Identifying Health Equity Factors That Influence the Public’s Perception of COVID-19 Health Information and Recommendations: A Scoping Review
Source: Int J Environ Res Public Health. 2022 Sep 23;19(19):12073. doi: 10.3390/ijerph191912073 (PMC9565967; doi:10.3390/ijerph191912073)
Supplement: Supplementary file 1 [file ijerph-19-12073-s001.zip › Supplementary S2- Final Proofread.pdf]

Appendices

Search Strategy

Ovid MEDLINE

Rylg#PHGOLQH+U,#DOO#4<79#wr#Mxo|#59/#5354A#

4#   khdowk#olwhudf|2# 9<:6#

5#   +khdowk#dgm5#+olwhudwh-#ru#olwhudf-,,1wl/de/hi1#   43<59#

6#   +frqvxphu-#ru#sxeolf#ru#sdwlhqw-,#dgm5#+hydoxdw-#ru#dvvhvv-#ru#dssudlv-#ru#mxgjh-#ru#mxgjlqj#
ru#fkrrvh-#ru#fkrvh#ru#fkrrvlqj-#ru#fkrlfh-#ru#ghflgh-#ru#ghflglqj#ru#xqghuvwdqg-#ru#xqghuvwrrg#
ru#frpsuhkhq-,,1wl/de/hi1#   5836<4#

7#   +Ghflvlrq-#dgm5#+dlg-#ru#wrro-#ru#vxssruw-#ru#jxlgh-#ru#pdnh-#ru#pdnlqj#ru#pdgh,,1wl/de/hi1
556868#

8#   Ghflvlrq#Pdnlqj2#   <<6:9#

9#   +lqirupdwlrq-#ru#fulwlf-,#dgm5#dssudlv-,1wl/de/hi1#47<<<#

:#   4#ru#5#ru#6#ru#7#ru#8#ru#9#$748<4#

;#   frqvxphu#khdowk#lqirupdwlrq2# 73;6#

<#   +frqvxphu-#dgm5#khdowk#lqirupdwlrq-,1wl/de/hi1# ;44#

43# +sdwlhqw-#ru#frqvxphu-#ru#sxeolf-#ru#phglf-#ru#khdowk-,#dgm7#+uhfrpphqgdwlrq-#ru#jxlgholqh-#
ru#jxlgdqfh-#ru#phdvxuh-#ru#dgylfh#ru#lqvwuxfwlrq-,,1wl/de/hi1#   5;:5<3#

44# jxlgholqhv#dv#wrslf2#dqg#+sdwlhqw-#ru#frqvxphu-#ru#sxeolf-,1wl/de/hi1# 45476#

45# ;#ru#<#ru#43#ru#44#   634875#

46# +frurqdyluxv2#ru#ehwdfrurqdyluxv2#ru#frurqdyluxv#lqihfwlrqv2,#dqg#+glvhdvh#rxweuhdnv2#ru#
hslghplfv2#ru#sdqghplfv2,# 73397#

47# +qFrY-#ru#534<qFrY#ru#4<qFrY#ru#FRYLG4<-#ru#FRYLG#ru#VDUV0FRY05#ru#VDUVFRY05#ru#
VDUVFRY5#ru#Vhyhuh#Dfxwh#Uhvsludwru|#V|qgurph#Frurqdyluxv#5#ru#Vhyhuh#Dfxwh#Uhvsludwru|#
V|qgurph#Frurqd#Yluxv#5,1wl/de/hi/qp/rw/r{/u{/s{1# 48763:#

48# +qhz#ru#qryho#ru#%4<%#ru#%534<%#ru#Zxkdq#ru#Kxehl#ru#Fklqd#ru#Fklqhvh,#dgm6#+frurqdyluxv-#
ru#frurqd#yluxv-#ru#ehwdfrurqdyluxv-#ru#FrY#ru#KFrY,,1wl/de/hi/rw1# 7:<75#

49# +frurqdyluxv-#ru#frurqd#yluxv-#ru#ehwdfrurqdyluxv-,#dgm6#+sdqghplf-#ru#hslghplf-#ru#
rxweuhdn-#ru#fulvlv,,1wl/de/hi/rw1#   ;;<3#

4:# +Zxkdq#ru#Kxehl,#dgm8#sqhxprqld,1wl/de/hi/rw1#687#

4;# 46#ru#47#ru#48#ru#49#ru#4:#   49364;#

4<# :#dqg#45#dqg#4;#########**533**

Cochrane Central Register of Controlled Trials (OVID)

HEP #Uhylhz v#0#Frfkudqh#Fhqwudo#Uhjlvwhu#ri#Frqwuroohg#Wuldov#?Mxqh#5354A#

#

4# khdow#dwwhudf|2# 736#

5# +khdow#dgm5#+dwwhudwh-#ru#dwwhudf-,1wl/de/nz1# 4;<8#

6# +frqvxphu-#ru#sxedf#ru#sdwlhqw-,#dgm5#hydoxdw#ru#dvvhvv-#ru#dssudlv-#ru#mxgjh-#ru#mxgjlqj# ru#fkrrvh-#ru#fkrvh#ru#fkrrvlqj-#ru#fkrlfh-#ghflgh-#ru#ghflglqj#ru#xqghuvwdqg-#ru#xqghuvwrrg# ru#frpsuhkhq-,1wl/de/nz1# ;:935#

7# +Ghflvlrq-#dgm5#dlg-#ru#wrro-#ru#vxssruw-#ru#jxlgh-#ru#pdnh-#ru#pdnlqj#ru#pdgh,1wl/de/nz1 # 4<8<4#

8# Ghflvlrq#Pdnlqj2# 563:#

9# +lqirupdwlrq-#ru#fulwlf-,#dgm5#dssudlv-,1wl/de/nz1# :9<#

:# 4#ru#5#ru#6#ru#7#ru#8#ru#9#43::<<#

;# frqvxphu#khdow#lqirupdwlrq2# 495#

<# +frqvxphu-#dgm5#khdow#lqirupdwlrq-,1wl/de/nz1#;9#

43# +sdwlhqw#ru#frqvxphu-#ru#sxedf-#ru#phglf-#ru#khdow-,#dgm7#+uhfrpphqgdwlrq-#ru#jxlghdqh-# ru#jxlgdqfh-#ru#phdvxuh-#ru#dgylfh#ru#lqvwuxfwlrq-,1wl/de/nz1# ;4876#

44# jxlghdqhv#dv#wrslf2#dqg#sdwlhqw#ru#frqvxphu#ru#sxedf-,1wl/de/nz1#479#

45# ;#ru#<#ru#43#ru#44# ;4;4;#

46# +frurqdyluxv2#ru#ehwdfrurqdyluxv2#ru#frurqdyluxv#lqihfwlrqv2,#dqg#+glvhdvh#rxweuhdnv2#ru# hslghplfv2#ru#sdqghplfv2,# 465#

47# +qFrY-#ru#534<qFrY#ru#4<qFrY#ru#FRYLG4<-#ru#FRYLG#ru#VDUV0FRY05#ru#VDUVFRY05#ru# VDUVFRY5#ru#Vhyhuh#Dfxwh#Uhvsludwru|#V|qgurph#Frurqdyluxv#5#ru#Vhyhuh#Dfxwh#Uhvsludwru|# V|qgurph#Frurqd#Yluxv#5,1wl/de/nz1#9459#

48# +qhz#ru#qryho#ru#%4<%#ru#%534<%#ru#Z xkdq#ru#Kxehl#ru#Fklqd#ru#Fklqhvh,#dgm6#+frurqdyluxv-# ru#frurqd#yluxv-#ru#ehwdfrurqdyluxv-#ru#FrY#ru#KFrY,,1wl/de/nz/rw1#5;38#

49# +frurqdyluxv-#ru#frurqd#yluxv-#ru#ehwdfrurqdyluxv-,#dgm6#+sdqghplf-#ru#hslghplf-#ru# rxweuhdn-#ru#fulvlv,,1wl/de/nz/rw1# 537#

4:# +Z xkdq#ru#Kxehl,#dgm8#sqhxprqld,1wl/de/nz/rw1# 78#

4;# 46#ru#47#ru#48#ru#49#ru#4:# 954<#

4<# :#dqg#45#dqg#4;######### **89**

APA Psycinfo

DSD#Sv|fLqir#?4;39#wr#Mxo|#Z hhn#6#5354A#

#

4# Khdow#Dwwhudf|2#667;#

5#   +khdowk#dgm5#+olwhudwh-#ru#olwhudf-,,1wz1#   79<7#

6#   +frqvxphu-#ru#sxeolf#ru#sdwlhqw-,#dgm5#+hydoxdw-#ru#dvvhvv-#ru#dssudlv-#ru#mxgjh-#ru#mxgjlqj#ru#fkrrvh-#ru#fkrvh#ru#fkrrvlqj-#ru#fkrlfh-#ghflgh-#ru#ghflglqj#ru#xqghuvwdqg-#ru#xqghuvwrrg#ru#frpsuhkhq-,,1wz1# 6:8<8#

7#   +Ghflvlrq-#dgm5#+dlg-#ru#wrro-#ru#vxssruw-#ru#jxlgh-#ru#pdnh-#ru#pdnlqj#ru#pdgh,,1wz1 #   466;;8#

8#   Ghflvlrq#Pdnlqj2#   :;53;#

9#   +Lqirupdwlrq-#ru#fulwlf-,#dgm5#dssudlv-,1wz1#   6:34#

:#   fulwlfdo#wklqnlqj2#6495#

;#   4#ru#5#ru#6#ru#7#ru#8#ru#9#ru#:# 4<6835#

<#   Khdowk#Lqirupdwlrq2#ru#Khdowk#Hgxfdwlrq2#   48<59#

43# +frqvxphu-#dgm5#khdowk#lqirupdwlrq-,1wz1#   546#

44# +sdwlhqw-#ru#frqvxphu-#ru#sxeolf-#ru#phglf-#ru#khdowk-,#dgm7#+uhfrpphqgdwlrq-#ru#jxlgholqh-#ru#jxlgdqfh-#ru#phdvxuh-#ru#dgylfh#ru#lqvwuxfwlrq-,,1wz1#   8:<8<#

45# <#ru#43#ru#44#   :5<<7#

46# +frurqdyluxv2#ru#ehwdfrurqdyluxv2#ru#frurqdyluxv#lqihfwlrqv2,#dqg#+glvhdvh#rxweuhdnv2#ru#hslghplfv2#ru#sdqghplfv2,# 4:5<#

47# +qFrY-#ru#534<qFrY#ru#4<qFrY#ru#FRYLG4<-#ru#FRYLG#ru#VDUV0FRY05#ru#VDUVFRY05#ru#VDUVFRY5#ru#Vhyhuh#Dfxwh#Uhvsludwru|#V|qgurph#Frurqdyluxv#5#ru#Vhyhuh#Dfxwh#Uhvsludwru|#V|qgurph#Frurqd#Yluxv#5,1wz1#   :549#

48# +qhz#ru#qryho#ru#%4<%#ru#%534<%#ru#Zxkdq#ru#Kxehl#ru#Fklqd#ru#Fklqhvh,#dgm6#+frurqdyluxv-#ru#frurqd#yluxv-#ru#ehwdfrurqdyluxv-#ru#FrY#ru#KFrY,,1wz1#4856#

49# +frurqdyluxv-#ru#frurqd#yluxv-#ru#ehwdfrurqdyluxv-,#dgm6#+sdqghplf-#ru#hslghplf-#ru#rxweuhdn-#ru#fulvlv,,1wz1#   99;#

4:# +Zxkdq#ru#Kxehl,#dgm8#sqhxprqld,1wz1#   :#

4;# 46#ru#47#ru#48#ru#49#ru#4:#   :69:#

4<# ;#dqg#45#dqg#4;########## **26**#

Embase#

Hpedvh#Fodvvlf.Hpedvh#?4<7:#wr#5354#Mxo|#59A##

4#   khdowk#olwhudf|2# 46;59#

5#   +khdowk#dgm5#+olwhudwh-#ru#olwhudf-,,1wl/de/hz1#   4764;#

6#   +frqvxphu-#ru#sxeolf#ru#sdwlhqw-,#dgm5#+hydoxdw-#ru#dvvhvv-#ru#dssudlv-#ru#mxgjh-#ru#mxgjlqj#ru#fkrrvh-#ru#fkrvh#ru#fkrrvlqj-#ru#fkrlfh-#ghflgh-#ru#ghflglqj#ru#xqghuvwdqg-#ru#xqghuvwrrg#ru#frpsuhkhq-,,1wl/de/hz1#   6<5:44#

7# +Ghflvlrq-#dqg#5#+dlg-#ru#wrro-#ru#vxssruw-#ru#jxlgh-#ru#pdnh-#ru#pdnlqj#ru#pdgh,,1wl/de/hz1

63;:94#

8# ghflvlrq#pdnlqj2# 583898#

9# +lqirupdwlrq-#ru#fulwlf-,#dgm5#dssudlv-,1wl/de/hz1# 4:;:7#

:# fulwlfdo#wklqnlqj2#5845#

;# 4#ru#5#ru#6#ru#7#ru#8#ru#9#ru#:# ;98546#

<# frqvxphu#khdowk#lqirupdwlrq2# 73:5#

43# +frqvxphu-#dgm5#khdowk#lqirupdwlrq-,1wl/de/hz1#<47#

44# +sdwlhqw-#ru#frqvxphu-#ru#sxeolf-#ru#phglf-#ru#khdowk-,#dgm7#+uhfrpphqgdwlrq-#ru#jxlgh-#ru#jxlgdqfh-#ru#phdvxuh-#ru#dgylfh#ru#lqvwuxfwlrq-,,1wl/de/hz1# 77439:#

45# <#ru#43#ru#44# 7786;6#

46# vduv0uhodwhg#frurqdyluxv2# 7:7#

47# +frurqdylulqdh2#ru#ehwdfrurqdyluxv2#ru#frurqdyluxv#lqihfwlrq2,#dqg#hslghplf2#ru#sdqghplf2,

43;39#

48# +qFrY-#ru#534<qFrY#ru#4<qFrY#ru#FRYLG4<-#ru#FRYLG#ru#VDUV0FRY05#ru#VDUVFRY05#ru#VDUV0FRY5#ru#VDUVFRY5#ru#Vhyhuh#Dfxwh#Uhvsludwru|#V|qgurph#Frurqdyluxv#5#ru#Vhyhuh#Dfxwh#Uhvsludwru|#V|qgurph#Frurqd#Yluxv#5,1wl/de/hz/kz/rw1# 487686#

49# +frurqdyluxv-#ru#frurqd#yluxv-#ru#ehwdfrurqdyluxv-,#dgm6#+sdqghplf-#ru#hslghplf-#ru#rxweuhdn-#ru#fulvlv,,1wl/de/hz/rw1# ;839#

4:# +Zxkdq#ru#Kxehl,#dgm8#sqhxprqld1wl/de/hz/rw1# 6<3#

4;# 46#ru#47#ru#48#ru#49#ru#4:# 489;73#

4<# ;#dqg#45#dqg#4;########**551**
